# Hedgehog and TGFβ signaling converge on Gli2 to control bony invasion and bone destruction in oral squamous cell carcinoma

**DOI:** 10.18632/oncotarget.12584

**Published:** 2016-10-12

**Authors:** Shellese A. Cannonier, Cara B. Gonzales, Kim Ely, Scott A. Guelcher, Julie A. Sterling

**Affiliations:** ^1^ Department of Veteran Affairs, Tennessee Valley Healthcare System, Nashville TN 37212, USA; ^2^ Center for Bone Biology, Vanderbilt University Medical Center, Nashville TN 37232, USA; ^3^ Department of Cancer Biology, Vanderbilt University Medical Center, Nashville TN 37232, USA; ^4^ Department of Comprehensive Dentistry, University of Texas Health Science Center San Antonio Dental School, San Antonio, TX 78229, USA; ^5^ Department of Pathology, Microbiology, and Immunology, Vanderbilt University Medical Center, Nashville TN 37232, USA; ^6^ Department of Chemical and Biomolecular Engineering, Vanderbilt University, Nashville TN 37235, USA; ^7^ Department of Biomedical Engineering, Vanderbilt University, Nashville TN 37235, USA; ^8^ Division of Clinical Pharmacology, Department of Medicine, Vanderbilt University Medical Center, Nashville TN 37232, USA

**Keywords:** oral cancer, Gli2, hedgehog, TGF-B, parathroid hormone related protein (PTHrP)

## Abstract

Oral Squamous Cell Carcinoma (OSCC) is the sixth most common cancer worldwide. OSCC invasion into the lymph nodes and mandible correlates with increased rates of recurrence and lower overall survival. Tumors that infiltrate mandibular bone proliferate rapidly and induce bone destruction. While survival rates have increased 12% over the last 20 years, this improvement is attributed to general advances in prevention, earlier detection, and updated treatments. Additionally, despite decades of research, the molecular mechanisms of OSCC invasion into the mandible are not well understood. Parathyroid Hormone-related Protein (PTHrP), has been shown to be essential for mandibular invasion in OSCC animal models, and our previous studies demonstrate that the transcription factor Gli2 increases PTHrP expression in tumor metastasis to bone. In OSCC, we investigated regulators of Gli2, including Hedgehog, TGFβ, and Wnt signaling to elucidate how PTHrP expression is controlled. Here we show that canonical Hedgehog and TGFβ signaling cooperate to increase PTHrP expression and mandibular invasion in a Gli2-dependent manner. Additionally, in an orthotopic model of mandibular invasion, inhibition of Gli2 using shRNA resulted in a significant decrease of both PTHrP expression and bony invasion. Collectively, our findings demonstrate that multiple signaling pathways converge on Gli2 to mediate PTHrP expression and bony invasion, highlighting Gli2 as a therapeutic target to prevent bony invasion in OSCC.

## INTRODUCTION

Oral Squamous Cell Carcinoma (OSCC) is a subset of head and neck squamous cell carcinomas (HNSCC), and accounts for the majority of cases [1, 2]. Known risk factors include smoking, alcohol, and chewing tobacco use [3]. Tumor resection remains the primary treatment for OSCC, although neoadjuvant and adjuvant therapies are often used [4]. These include radiation therapy, chemotherapy, neck dissection to remove lymph node metastases, and most recently, the EGFR inhibitory antibody, Cetuximab [5, 6]. These invasive tumors infiltrate cervical lymph nodes and the mandible, which is significantly correlated with increased local recurrence and decreased overall survival [7]. Tumors that invade into the mandible disrupt normal bone remodeling, causing large amounts of bone destruction, chronic pain, and impairs eating and speaking abilities. These patients require surgical resection of the tumor-laden bone, which includes mandible reconstruction, followed by radiation therapy and chemotherapy [8]. Unfortunately, OSCC patients have a 30-40% local recurrence rate, which leads to significantly reduced survival rates [9].

A syngeneic model of oral cancer demonstrated that PTHrP is required for OSCC invasion into the mandible [10]. PTHrP, a known regulator of tumor-induced bone disease, is expressed at low levels in healthy adult tissues, and thus it is a potential target for drug treatment [11, 12]. Unfortunately, the molecular mechanisms that control PTHrP expression in OSCC are not known. In long bone formation, PTHrP is regulated by canonical Hedgehog (Hh) signaling, where receptor proteins, Smoothened (Smo) and Patched (Ptch) control Gli protein and downstream target genes. However, in the context of cancer, Gli can also be regulated by non-canonical Hh signaling [13], through direct Gli activation stemming from AKT, MEK, or S6K1 activity [14, 15]. Additionally, Gli can be regulated transcriptionally through other signaling pathways, where TGFβ and Wnt signaling can increase expression and induce activation of Gli2 independent of its upstream regulators [16, 17]. In OSCC, PTHrP expression can be stimulated by activation of Gli2 using TGFβ and our clinical data shows that Gli2 expression correlates with bony invasion. Here, we demonstrate that PTHrP expression is regulated by Gli2 and both Gli2 activity and PTHrP expression are controlled concomitantly through Hh and TGFβ signaling. Using an orthotopic *in vivo* model, we validate that bony invasion and bone destruction are regulated by PTHrP through modulation of Gli activity.

## RESULTS

### PTHrP mRNA levels predict bony invasion and bone destruction *in vivo*

**Figure 1:**
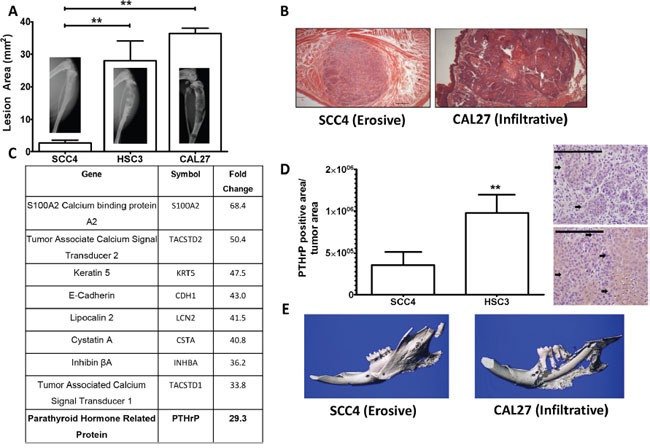
PTHrP mRNA levels predict bony invasion and bone destruction in two mouse models **A.** Tumor model of bone destruction. X-ray data of SCC4 cells injected into the tibiae of athymic mice show significantly less bone destruction than HSC3 and CAL27 cells. **B.** H&E stained histological sections of SCC4 and CAL27 tumors taken from tip-of-the-tongue injections in athymice mice show morphological differences. SCC4 cells bear an erosive phenotype, while CAL27 cells bear an infiltrative phenotype. **C.** PTHrP is upregulated almost 30-fold in bony invasive OSCC. The top ten upregulated genes as determined from a genome wide microarray study comparing mRNA levels of CAL27 cells (bony invasive OSCC) vs SCC4 (non-bony OSCC). PTHrP is highlighted because it is known to be essential for OSCC bony invasion. **D.** PTHrP levels correlate with bony invasion status. SCC4 cells injected into the tibiae of athymic mice show low PTHrP staining by IHC, while HSC3 cells show significantly larger amounts of PTHrP expression, as denoted by the black arrows. (Images at 40X, scale bar is 200μm) **E.** Orthotopic model of bony invasion. Representative μCT scans of mandibles dissected from mice bearing tumors from masseter muscle injections. SCC4 cells show minimal bone destruction and small amounts of new bone formation, while CAL27 cells show extensive bone destruction.

OSCC commonly invades with an erosive or infiltrative pattern. To discern between soft tissue invasion potential and bony invasion potential we utilized a well-established model of bone destruction, where tumor cells are inoculated directly into the tibia and allowed to establish for four weeks. Mice are then sacrificed and tumor burden and trabecular bone loss quantified. Using this model, we injected three human OSCC cell lines (SCC4, CAL27, and HSC3) and found that SCC4 cells showed minimal bone destruction. In contrast, CAL27 and HSC3 cells showed significantly more bone destruction (Figure 1A) while tumor burden remained similar to SCC4 cells (Supplementary Figure S1A and S1B).

To identify differences between OSCC cells capable of inducing destruction of mandibular bone compared to those that are incapable, we utilized a genome-wide microarray comparing SCC4 and CAL27 cells (Figure 1B) to identify genes associated with bone destruction. SCC4 and CAL27 cells were derived from Caucasian males, who have the highest incidence of OSCC in the United States [2]. Additionally, the site of origin for both cell lines were the tongue, correlating well with the finding that a considerable number of patients with OSCC of the tongue develop mandibular invasion [18]. Our micro-array analyses identified genes over-expressed in the CAL27 cells compared to the SCC4 cells, with several genes being involved in calcium signaling. Of notable interest was Parathyroid Hormone-related Protein (PTHrP) which showed 30-fold higher expression in bony invasive CAL27 cells (Figure 1C). PTHrP and Gli2 expression levels in CAL27 and SCC4 cells were verified using qRT-PCR along with eight additional OSCC cell lines. Nine of the ten cell lines tested expressed Gli2 and PTHrP (Supplementary Figure S2A & S2B). To verify these differences *in vivo*, we used immunohistochemical staining to measure PTHrP and found that PTHrP secretion significantly correlated with the bony invasive HSC3 cells, while SCC4-injected tibiae showed very little PTHrP secretion (Figure 1D and Supplementary Figure S3).

In addition to bone destruction, PTHrP levels can also predict bony invasion [19]. To verify this correlation *in vivo*, we used an orthotopic model of bony invasion, where tumor cells are injected into the masseter muscle adjacent to the mandible and directly invade into the surrounding tissues. SCC4 cells have minimal destructive effect on mandibular bone, while CAL27 cells induce significant amounts of bone loss (Figure 1E). These findings correlate OSCC PTHrP levels with bony invasion and bone destruction in two separate models.

### OSCC express PTHrP in a Gli2-dependent manner

**Figure 2:**
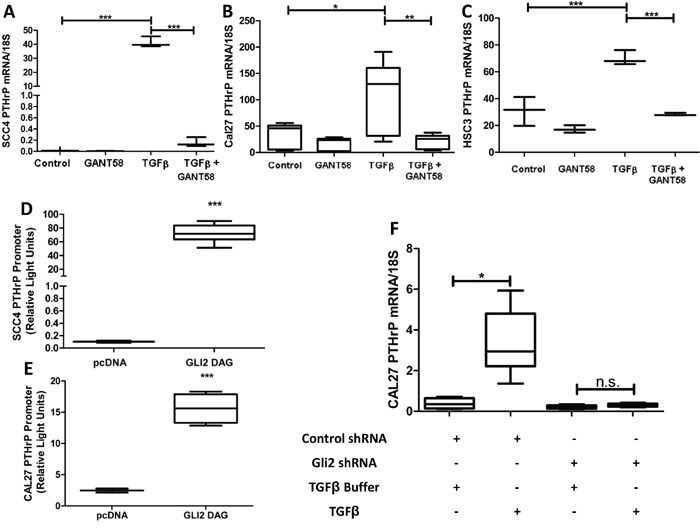
OSCC express PTHrP in a Gli2 dependent manner **A-C.** Gli2 inhibition decreases basal PTHrP expression and suppresses TGFβ induced PTHrP expression. qRT-PCR was used to determine PTHrP mRNA levels of three OSCC cell lines (CAL27, SCC4, and HSC3) that were treated with 10uM of the Gli inhibitor, GANT58 or the solvent control DMSO, with or without the addition of 10ng/ml TGFβ, which induces PTHrP expression. In all groups GANT58 treatment significantly decreased PTHrP expression. **D**&**E.** Gli2 over-expression significantly increases PTHrP expression. SCC4 and CAL27 cells were co-transfected to overexpress Gli2 SA662 Flag (Gli2 protein resistant to ubiquitination based proteosomal degradation), as well as a PTHrP firefly luciferase reporter plasmid and a constitutively active Renilla luciferase reporter plasmid. 48 hours later cells were harvested and firefly activity quantified. In both lines, Gli2 over-expression increased PTHrP. **F.** shRNA mediated Gli2 silencing prevents TGFB induced PTHrP expression. qRT-PCR was used to determine PTHrP mRNA levels in CAL27 cells transfected with non-coding hairpins or a pool of four independent hairpins against Gli2. Both groups showed low basal expression of PTHrP, but when treated with TGFB, control cells significantly increased PTHrP mRNA expression while Gli2 deficient cells were unable to increase PTHrP mRNA.

We evaluated the contribution of Gli2 to regulation of PTHrP expression in OSCC using Gli Antagonist 58 (GANT58), a small molecule inhibitor specific to Gli [20]. Gli2 inhibition significantly decreased PTHrP expression at both basal and stimulated levels in several OSCC lines (Figure 2A–2C). Additionally, Gli2 is sufficient to increase PTHrP expression, as Gli2-SA662 over-expression induced by plasmids significantly increased PTHrP expression (Figure 2D & 2E) [21]. We observed that wild-type over-expression Gli plasmids does not lead to increased PTHrP promoter activity (Supplementary Figure S4A), but because Gli2 expression is undetectable in many adult tissue compartments (except for basal/stem cell compartments), we surmise that cells with Gli2 activity have reactivated normally silenced signaling mechanisms, highlighting its potential importance in OSCC tumorigenesis [22]. To determine if Gli2 is required for PTHrP expression, we used shRNA to stably knock down Gli2 expression. Loss of Gli2 prevented stimulated PTHrP expression (Figure 2F). Together, our findings suggest that Gli2 modulates PTHrP and is required for its expression.

### Hedgehog receptor signaling is required for Gli2 activity and PTHrP expression

**Figure 3:**
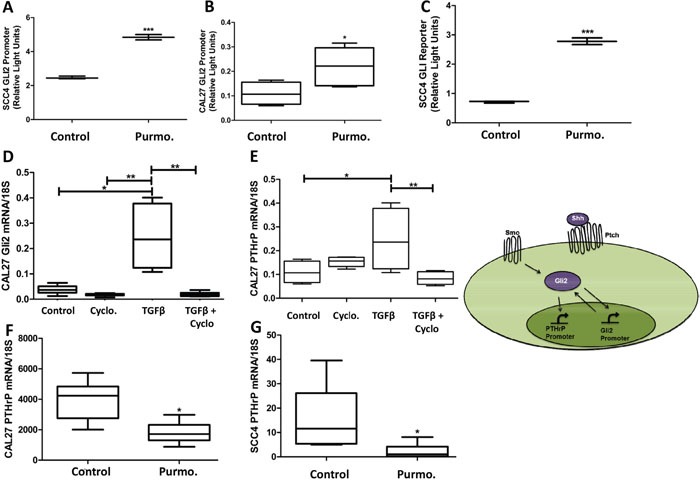
Hedgehog receptor signaling is required for Gli2 activity and PTHrP expression but alone is not sufficient for PTHrP expression **A**&**B.** Hh stimulation increases Gli2 promoter activity. OSCC cells were transfected using luciferase based reporter plasmids to assay endogenous Gli2 promoter activity and then stimulated with 10uM purmorphamine, resulting in a significant increase in Gli2 promoter activity. **C.** Hh stimulation increases Gli protein activity. OSCC cells were transfected using luciferase based reporter plasmids to assay Gli2 protein activity, and then stimulated with purmorphamine, resulting in a significant increase in Gli2 protein activity. **D**&**E.** Canonical Hh signaling is required for both Gli2 and PTHrP expression. qRT-PCR was used to determine PTHrP mRNA levels of CAL27 cells that were treated with 12nM of cyclopamine, or the solvent control DMSO, with or without the addition of TGFβ. Cyclopamine decreased basal PTHrP expression as well as significantly inhibited TGFβ-induced PTHrP expression. Inset demonstrates the dual role of Gli2 protein to increasing Gli2 and PTHrP expression. **F**&**G.** Hh stimulation is not sufficient to increase PTHrP expression. qRT-PCR was used to determine PTHrP mRNA levels of CAL27 cells that were treated with purmorphamine, which did not induce PTHrP expression, but instead decreased basal levels of PTHrP, suggesting a possible feedback loop.

As the canonical regulator of Gli2, we examined the role of receptor based Hh signaling on regulating Gli2 activity and PTHrP expression using purmorphamine, a Smo-specific agonist, or cyclopamine, a Smo-specific antagonist. We demonstrate that purmorphamine significantly increased Gli2 promoter activity (Figure 3A & 3B) as well as Gli2 protein activity using luciferase-based reporter assays (Figure 3C). As expected, cyclopamine significantly decreased stimulated Gli2 expression (Figure 3D) and prevented an increase of stimulated levels of PTHrP, highlighting the requirement of canonical Hh signaling for PTHrP expression (Figure 3E). Unexpectedly, purmorphamine did not significantly increase PTHrP expression (Figure 3F & 3G) and activation of Smo using Indian hedgehog protein (Ihh) showed similar results (Supplementary Figure S4B). Collectively, these observations suggest that Hh signaling is necessary, but not sufficient for PTHrP expression, and thus other pathways are required.

### TGFβ signaling modulates Gli2 and PTHrP expression

**Figure 4:**
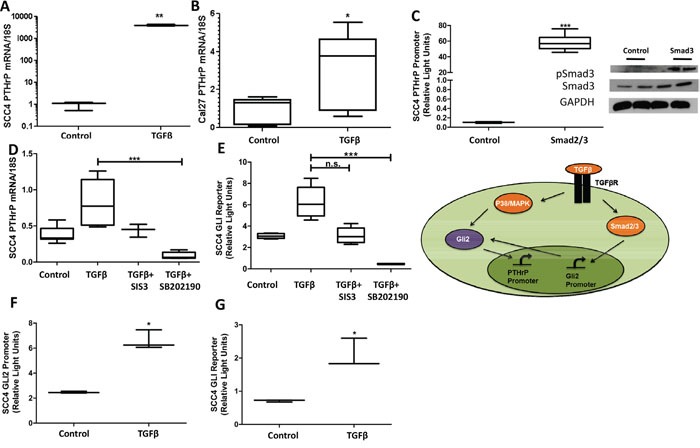
TGFβ signaling modulates Gli2 and PTHrP expression **A&B.** TGFβ signaling increased PTHrP expression.qRT-PCR was used to determine PTHrP mRNA levels of SCC4 and CAL27 cells that were treated with TGFβ or the buffer control. TGFβ treatment significantly increased PTHrP in all cell lines tested. **C.** Smad2/3 over-expression increased PTHrP expression. SCC4 cells were co-transfected to overexpress equal amounts of Smad2 and Smad3 (see insert for protein confirmation by Western blot), as well as a PTHrP firefly luciferase reporter plasmid and a constitutively active Renilla luciferase reporter plasmid. 48 hours after transfections, cells were harvested and firefly activity quantified. Smad over-expression significantly increased PTHrP promoter expression. **D**&**E.** Canonical and non-canonical TGFβ inhibition decreased Gli activity and PTHrP expression. qRT-PCR was used to determine PTHrP mRNA levels of SCC4 cells treated with TGFβ, and SIS3, a Smad3 inhibitor, or SB202190, a p38/MAPK inhibitor. While Smad3 inhibition trended to significantly decrease Gli activity and PTHrP expression, only p38/MAPK inhibition significantly decreased both. **F**&**G.** TGFβ signaling increased Gli2 promotor and protein activity. SCC4 cells were co-transfected with an endogenous Gli2 promoter construct, or a Gli2 protein reporter construct, as well as a constitutively active Renilla luciferase reporter plasmid. 24 hours after transfections, cells were treated with TGFβ or the buffer control and harvested 24 hours later before firefly activity was quantified. Both promoter and protein activity of Gli2 was significantly increased with TGFβ signaling. Inset demonstrates the dual role TGFβ signaling has on increasing Gli2 at the level of mRNA as well as protein.

Studies on breast cancer metastasis to bone highlight the importance of PTHrP for regulation of bone destruction by a non-canonical Gli2-dependent mechanism through TGFβ signaling [23, 24]. Several lines of evidence support TGFβ signaling induction of Gli, independent of Hh signaling [17, 25, 26]. We examined the role of TGF-β on the regulation of Gli2 and PTHrP and found that TGFβ stimulation significantly increased levels of PTHrP expression (Figure 4A & 4B). Moreover, increased PTHrP expression is facilitated through canonical TGFβ signaling, since the use of Smad protein over-expression plasmids led to a marked increase in PTHrP expression (Figure 4C). Inhibition of canonical TGFβ signaling using the Smad3 inhibitor, SIS3, decreased PTHrP expression (but not significantly), and inhibition of non-canonical TGFβ signaling using the p38/MAPK inhibitor, SB202190, significantly decreased Gli activity and PTHrP expression (Figure 4D & 4E). Importantly, the TGFβ mediated increased of PTHrP expression correlates with an increase in both Gli2 promoter and protein activity levels (Figure 4F & 4G).

### Intracellular Wnt signaling increases PTHrP expression through crosstalk with Hh signaling

**Figure 5:**
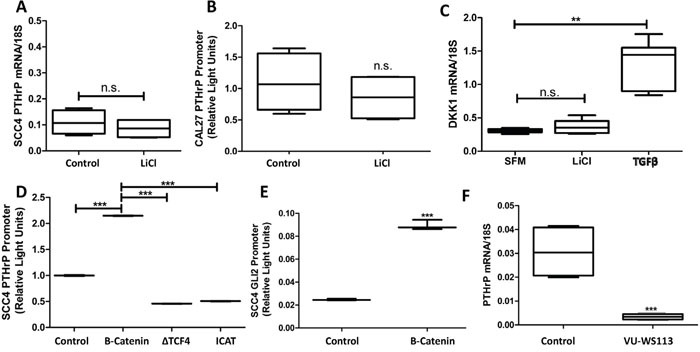
Intracellular Wnt signaling increases PTHrP expression through crosstalk with Hh signaling **A&B.** Wnt signaling activation is insufficient to increase PTHrP expression.SCC4 cells were treated with LiCL and PTHrP mRNA measured by qRT-PCR. Li Cl treatment does not significantly increase PTHrP expression. Similar results were seen for PTHrP promoter activity by dual-luciferase assays in CAL27 cells treated with LiCl for 24hours. **C.** Canonical Wnt signaling is not active in OSCC. CAL27 cells were treated with Li-Cl, TFGβ, or control for 24 hours before being harvested for qRT-PCR. Active Wnt signaling, indicated by higher levels of the β-catenin target gene DKK1, is observed with TFGβ but not Li-Cl stimulation. **D.** Intracellular Wnt signaling modulates PTHrP expression. SCC4 cells were transfected to express a PTHrP firefly luciferase reporter and a constitutively active Renilla luciferase reporter plasmid in combination with a β-catenin protein expressing plasmid, a dominant negative TCF4 protein expressing plasmid, or an ICAT protein expressing plasmid. Intracellular Wnt activation using β-catenin led to a significant increase in PTHrP promoter activity; while inhibitors of β-catenin decreased PTHrP promoter activity **E.** β-catenin over-expression increases Gli2 promoter activity. SCC4 cells were transfected to express an endogenous Gli2 promoter firefly luciferase reporter and a constitutively active Renilla luciferase reporter plasmid in combination with a β-catenin protein expressing plasmid. 48 hours after transfection, cells were harvested and firefly activity quantified, which demonstrated a significant increase in Gli2 promoter activity, quite similar to that of PTHrP promoter activity. **F.** Intracellular Wnt inhibition decreases PTHrP expression. qRT-PCR was used to determine PTHrP mRNA levels of CAL27 cells that were treated with or without 10uM of the CK1 inhibitor, VU-WS113. Treatment with VU-WS113 significantly decreased PTHrP expression.

Wnt signaling plays an essential role in bone biology, and research has demonstrated its significance in supporting metastases to bone [27, 28]. We explored the role of Wnt signaling in OSCC on increasing PTHrP expression using Lithium Chloride (LiCl) to inhibit activity of glycogen synthase kinase-3β (GSK-3), a negative regulator of Wnt signaling. Wnt activation was found to have no effect on PTHrP expression (Figure 5A & 5B). Additionally, qRT-PCR for the Wnt target gene, DKK1, showed no significant changes in expression between control and LiCl-stimulated cells (Figure 5C), suggesting that canonical Wnt signaling is inactive. Since Wnt signaling may play a role in PTHrP expression downstream of GSK-3, we evaluated the role of β-catenin, the downstream effector protein, on PTHrP expression. We found that β-catenin over-expressing plasmids increased PTHrP expression levels. PTHrP levels decrease when two β-catenin inhibitory proteins, dominant negative TCF4 and ICAT, are over-expressed (Figure 5D). There are several β-catenin/TCF4 binding sites on the promoter region of Gli2 [29], so we tested the role of β-catenin on increasing Gli2 promoter activity using luciferase-based reporter assays and found that β-catenin significantly increased Gli2 promoter activity (Figure 5E). We tested the requirement of intracellular Wnt signaling for PTHrP expression using the small molecule Wnt inhibitor VU-WS113, which led to a significant reduction in stimulated levels of PTHrP (Figure 5F). Surprisingly, direct inhibition of Wnt signaling at the receptor level using Sclerostin significantly increased PTHrP expression (Supplementary Figure S5A), which highlights abnormal Wnt signaling and indicates possible downstream crosstalk with Gli. Indeed, indirect modulation of Wnt signaling using LiCl and VU-WS113 have been shown to affect Hh signaling as reported [30]. Together, our data support that Wnt signaling in OSCC is irregular; suggesting that only Wnt activation at the level of β-catenin consistently modulates PTHrP expression, where Gli2 expression is also increased.

### Gli2 expression is required to increase PTHrP expression, bony invasion and bone destruction

**Figure 6:**
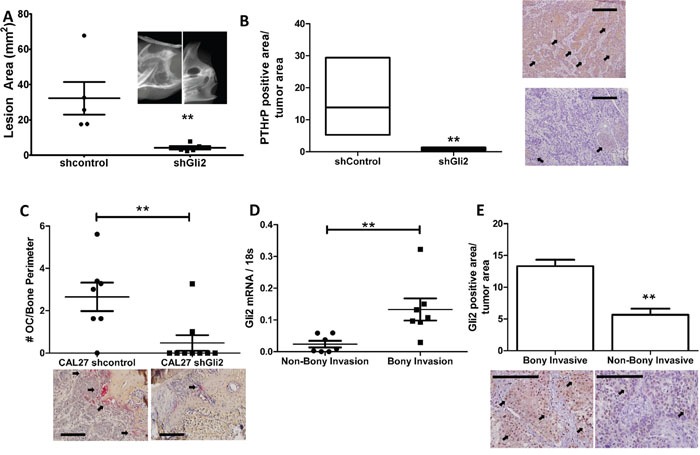
Gli2 is required to increase PTHrP expression, bony invasion and bone destruction **A.** Loss of Gli2 using shRNA decreases bone destruction *in vivo*. CAL27 cells stably expressing shRNA against a non-silencing control sequence or Gli2 were injected into the masseter muscle of male athymic mice and allowed to progress for eight weeks. Mice were then sacrificed and mandibles dissected for high resolution x-rays. Significantly less bone destruction, as measured by lesion area, was observed in the shGli2 group. **B.** Loss of Gli2 decreases PTHrP levels *in vivo*. CAL27 tumors expressing shRNA against Gli2 show significantly lower PTHrP protein levels by IHC. (Inset is 40X with 200μm scale bars. Arrows denote positive staining) Loss of Gli2 expression is verified using IHC. **C.** Loss of Gli2 decreases osteoclasts numbers *in vivo*. Tartrate Resistant Acid Phosphate (TRAP) staining was used to identify osteoclasts at the tumor bone interface of mandible sections. We found a significantly larger number of TRAP-positive multinucleated cells in the control group as compared to the shGli2 group. **D.** Gli2 mRNA is associated with bony invasion in clinical samples. qRT-PCR was done on human OSCC clinical samples, where patients that underwent a mandiblectomy were classified as bony invasive (BI), while samples from patients who did not undergo amandiblectomy were classified as non-bony invasive (NBI). Samples from the BI group had significantly higher expression of Gli2. **E.** Gli2 protein correlates with bony invasion in human clinical samples. A larger cohort of clinical samples based on the selection criteria described above was used for IHC against Gli2. We show that Gli2 protein also significantly correlated with bony invasion.

To validate that Gli2 is required for bony invasion, we utilized shRNA to stably knock down expression of Gli2 in bony invasive CAL27 cells (Supplementary Figure S6A). Non-silencing control cells and shGli2 cells were injected into the masseter muscle of male athymic mice and allowed to progress for eight weeks. While both groups developed tumors, control tumors had significantly increased levels of bony invasion as well as bone destruction (Figure 6A). Surprisingly, loss of Gli2 led to smaller tumors, some of which were unable to invade into surrounding tissues. CAL27 shGli2 tumors had significantly lower PTHrP protein levels as determined by IHC (Figure 6B images) and histological data confirmed loss of Gli2 protein in the tumors (Supplementary Figure S6A images). Additionally, Tartrate Resistant Acid Phosphatase (TRAP staining) confirmed a significantly smaller numbers of osteoclasts at the tumor-bone interface in mandibular sections (Figure 6C).

### Gli2 levels correlate with bony invasion in clinical OSCC samples

OSCC samples from patients were characterized as bony invasive if patients underwent a mandiblectomy or non-bony invasive if patients only underwent soft tissue surgery. Eight samples of both bony invasion and non-bony invasion were used to measure Gli2 mRNA expression levels. By qRT-PCR, patients with bony invasion had significantly higher expression of Gli2 (Figure 6D). Additionally, immuno-histochemical staining of a larger cohort of paraffin embedded OSCC samples showed similar results for Gli2 protein, where Gli2 levels were significantly up-regulated in bony invasive samples (Figure 6E). These findings are clinically relevant, as Gli2 and TGFβ1 are significantly co-expressed in a cohort of 279 head and neck carcinomas catalogued in cBioPortal (Supplementary Figure S6B) [31].

## DISCUSSION

**Figure 7:**
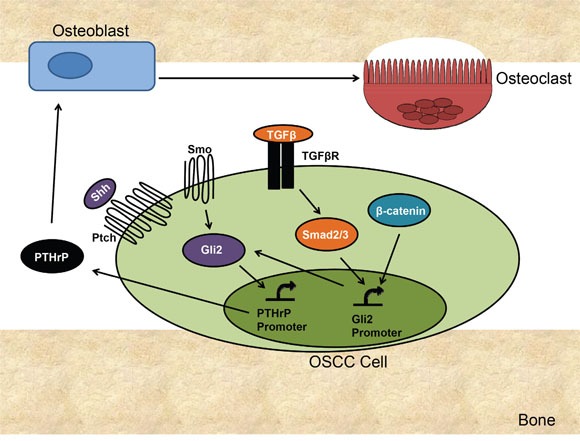
Proposed Mechanism of Gli2 Mediated Bone Destruction in OSCC OSCC signals through several signaling pathways to increase PTHrP. Canonical Hh signaling activates Gli2 protein accumulation and increases Gli2 expression, both of which can increase PTHrP expression. TGFβ signaling increases Smad2/3 activation, which increases Gli2 expression. Increased p38/MAPK activity can also increase Gli2 levels by stabilizing Gli2 protein. B-catenin activation increases Gli2 expression and directly and may also indirectly affect Gli2 protein levels through cross-talk. Gli2 increases PTHrP expression in OSCC, which results in an increase in osteoblast-mediated (through RANKL/RANK) osteoclast activation, resulting in increased bone resorption.

We have demonstrated that in OSCC, Gli2 is the central regulator of PTHrP expression (Figure 7). Importantly, we have identified several signaling pathways that control Gli2 at the level of mRNA expression and protein activity. Gli2 directly correlates with PTHrP expression and is both necessary and sufficient for PTHrP expression. Along with Hh signaling, we have identified TGFβ signaling as a major contributor to Gli activation and we show that both pathways are important for increasing Gli levels and thus PTHrP expression. We have also identified Wnt signaling as an activating pathway for Gli, although this seems to be driven in a non-canonical manner. Targeting Gli2 in OSCC using shRNA significantly decreased PTHrP expression and prevented bony invasion and bone destruction *in vivo*. Finally, our clinical data showing that Gli2 levels correlate with bony invasion strongly supports our findings that Gli2 controls PTHrP expression in OSCC.

Canonical Hh signaling is a highly conserved signaling pathway essential for normal development in higher organisms. In adults, Hh signaling is silenced in almost all tissues, but is often reactivated in tumorigenesis. Basal cell carcinoma and medulloblastoma are Hh-driven tumors, where loss of Ptch leads to constitutive activation of Hh signaling [32]. Additionally, there are reports of canonical and non-canonical Hh signaling in other tumor types, such as breast, lung, prostate, and colon, where Hh signaling is not the driving mutation but is involved in tumor progression and contributes to treatment failure and tumor recurrence [13, 33]. There have also been several published findings on Hh signaling in OSCC, where it is known that Hh signaling contributes to growth, migration and invasion [34, 35]. In our system, OSCC signals through both canonical and non-canonical Hh signaling to increase Gli2 activation and expression of downstream target genes. Alternatively, both TFGβ and Wnt signaling pathways activate transcription of Gli2, and we show that TFGβ increases Gli2 protein activity as well. Our findings highlight the complexity of Gli regulation and PTHrP expression in OSCC and demonstrate the importance of inhibiting Hh signaling at the level of Gli, as compared to upstream receptor-based inhibition.

The contribution of Wnt signaling to Gli2 and PTHrP activation is also complex. While Wnt signaling in OSCC does not appear to be normal, β-catenin activation can modulate PTHrP expression, and this activation occurs via Gli2. The inability of cells to respond to Wnt stimulation led us to believe that Wnt signaling was not activated in these cells, thus, we found it surprising that Wnt inhibition could decrease PTHrP expression. We investigated if this inhibition was independent of Gli2 using qRT-PCR and found that VU-WS113 treatment significantly decreased Gli2 protein activity (Supplementary Figure S5B), supporting the concept of crosstalk between the two pathways. Indeed, the mechanism of Wnt inhibition using VU-WS113 is through inhibition of CK1, an inhibitory kinase of β-catenin that has been shown to be a potent inhibitor of Hh signaling [30]. These findings are in line with reports of crosstalk between canonical Hh and Wnt signaling, which has been documented in normal development, wound repair and tumorigenesis [16, 36, 37]. Additionally, inhibition of Wnt signaling at the receptor level using Sclerostin increased PTHrP expression, suggesting that while β-catenin activation increases Gli2 and PTHrP expression, upstream activation of Wnt signaling has an opposite effect. These results are consistent with other literature showing that Hh and Wnt signaling can function as negative regulators of one another [38]. Thus, our data strongly suggests that Wnt regulation of PTHrP occurs at least in part, via Gli2.

In several OSCC lines tested, TGFβ stimulation led to an increase in PTHrP expression, with a similar increase in Gli2 transcription and protein activity. This was found to be mediated in a Smad-dependent manner. As there are several published reports demonstrating that the Smad binding sites on the Gli2 promoter are functional, we surmise that Gli2 is a direct target of TGFβ signaling [28, 29, 39]. The contribution of non-canonical TGFβ signaling on increasing Gli2 and PTHrP expression is also in line with other published findings, where several downstream targets of p38/MAPK including MEF2C and NF-kβ can induce expression of Hh ligands as well as Gli [13, 40]. Our group has published on TGFβ mediated activation of Gli2 in other tumor types that do not express the conical Hh signaling receptors, Smo and Ptch. We have found however, that in OSCC, canonical Hh signaling is required for TGFβ stimulation to increase Gli activity and PTHrP expression, suggesting a cooperative or synergistic signaling system.

Taken together, our data demonstrates a complex yet interconnected signaling network controlling PTHrP expression. Importantly, these mechanisms all converge on Gli2, highlighting its importance on regulating PTHrP expression. Previous findings have highlighted the importance of PTHrP for bony invasion in murine OSCC, but until now, the mechanism controlling PTHrP expression remained unknown [10]. Other studies have shown the prognostic value of PTHrP expression for predicting bony invasion using human samples, and we have built upon these findings to both identify and test regulators of PTHrP. It is important to note that Gli2 regulation of PTHrP is observed in other tumors. In tumors that metastasize to bone, PTHrP plays an important role in promoting tumor progression and bone destruction [24, 41, 42]. In a process coined “The Vicious Cycle”, metastatic tumor cells arrive at the bone microenvironment and secrete factors that directly or indirectly lead to excessive osteoclast-mediated bone resorption. In bone metastatic MDA-MB-231 breast cancer cells, PTHrP expression has been shown to be regulated by non-canonical Hh signaling [23]. Similar to OSCC, these cells increase Gli2 levels in response to TGFβ, which leads to an increase in PTHrP. Dissimilar to OSCC however; PTHrP in breast cancer cells can also be modulated by classical Wnt signaling [28]. Additionally, bone metastatic MDA-MB-231 cells do not express canonical Hh receptor signaling proteins, nor do they respond to Hh receptor based inhibition or activation, highlighting the complexity of PTHrP and Gli2 regulation in different systems [23].

OSCC invasion into the mandible is a serious occurrence that significantly impacts patient overall survival and quality of life [43]. These patients have high rates of recurrence at or near the site of the original tumor, emphasizing the need for more effective anti-tumor therapies. Currently, the only FDA approved targeted treatment for OSCC is Cetuximab, a monoclonal antibody against EGFR [44]. While Cetuximab significantly increases overall survival in patients with locally advanced disease, patients with recurrent and/or metastatic disease only survive three months longer when Cetuximab is added to their treatment regimen [45, 46]. Thus, it is clear that other targets are needed. PTHrP expression significantly correlates with bone involvement in OSCC patient samples and previous studies have identified PTHrP as an essential component of bony invasion and bone destruction in OSCC [10, 19]. However, limited published work on the mechanism of PTHrP regulation in OSCC exists. Through this study, we have identified key signaling mechanisms that control PTHrP-mediated bony invasion. The contributions of Hh, TGFβ, and Wnt signaling on Gli2 strongly suggest that bony invasion is regulated in a multifaceted system. The variability of receptor signaling control among OSCC cell lines highlights the importance of targeting the common downstream mediator, Gli2. Our work highlights mechanisms that can contribute to the observed clinical failures of Smo inhibitors, and we have demonstrated the value of targeted downstream Gli inhibitors to prevent treatment resistance and disease recurrence [47]. We have provided sufficient evidence to support that Gli2 controls bony invasion and bone destruction in OSCC via regulation of PTHrP. Our findings highlight the feasibility of using Gli2 inhibitors to prevent mandibular invasion and bone destruction in OSCC patients and should be evaluated further as a possible adjuvant treatment.

## MATERIALS AND METHODS

### Cell lines

The OSCC cell lines, SCC4, CAL27, and HSC3 were provided by Cara Gonzales at UTHSCSA, and were purchased from American Type Culture Collection (ATCC). The remaining OSCC cell lines, SCC9, SCC15, SCC25, SCC61, SCC131, SCC1352, and VU1729 were donated by Dr. Stephen Brant at Vanderbilt University. Cell lines were tested for mycoplasma and if positive, were treated with BM Cyclin (Roche). Cell lines were cultured in 50% Dulbecco's modification of Eagle's medium and 50% Nutrient Mixture F12 (DMEM/F12) (ThermoFisher Scientific), supplemented with 10% fetal bovine serum (FBS) (Hyclone Laboratories) and 1% penicillin/streptomycin (Mediatech). Cells were maintained at 37°C with 5% CO_2_.

### Genome wide microarray

Total RNA was extracted from OSCC cells stimulated with TGFβ or the buffer control using RNA Stat 60 reagent (AMSBIO) according to the manufacturer's protocol. The RNA was sent to the UTHSCSA Microarray Core facility and used as a template for double-stranded cDNA synthesis, followed by biotin-labeled cRNA synthesis. The cRNA was fragmented and hybridized to the U133A GeneChips overnight at 45°C in a rotating incubator. Hybridized cRNA was fluorescently labeled with anti-biotin antibodies and streptavidin phycoerythrin dye conjugate on a programmable microfluidics workstation. The probe arrays were scanned twice and the stored images were analyzed using the GeneChip MAS 5.0. Signal intensities were normalized and scaled using MAS 5.0 for comparison analysis of experimental and baseline arrays. Significantly up-regulated and down-regulated genes were identified by MAS 5.0.

### Drug treatments

All drug treatments were carried out in serum free DMEM/F12 for 24 hours. TGFβ and Sclerostin (R&D Systems) were used at 10ng/ml and 1-2ug/ml respectively. TGFβ buffer (5%BSA in 4mmol HCl) was used as a control. GANT58 and Lithium Chloride (Sigma-Aldrich) were used at 10μM and 20mM respectively. Purmorphamine and SIS3 (EMD Millipore) were used at 10μM. Cyclopamine (LC Labs) was used at 12nM. SB202190 (Tocris) was used at 10μM. VU-WS113, a less cytotoxic derivative of Pyrvinium, was a gift from Dr. Ethan Lee at Vanderbilt University.

### Transfections

Cells were transfected per manufacturer's instructions using Lipofectamine 2000 (ThermoFisher Scientific). Briefly, cells were incubated overnight in Opti-Mem (ThermoFisher Scientific) before being transfected at a ratio of 1.25 ug DNA to 1ul Lipofectamine 2000. Fresh media (complete with FBS and antibiotics) was added to cells the following morning. Transiently transfected cells were harvested 48 hours after transfections, while stably transfected cells were maintained in antibiotic media. 400ug/ml G418 or 125 ng/ml puromycin were used for two weeks to select for transfected cells. Thereafter, cells were cultured in antibiotic maintenance media, which was 200ug/ml G418 or 62.5 ng/ml puromycin.

### Plasmids for stable transfections

Gli2-SA662 Flag was a gift from Vladimir Spiegelman from the University of Wisconsin Medical School [21]. The Gli2 protein produced from these plasmids harbor a single point mutation, changing the serine at amino acid position 662 to alanine. This enables resistance to proteasomal degradation by ubiquitination [21]. Three pooled shRNA constructs against Gli2 and the non-silencing control (Origene) were used to knock-down Gli2 expression. DNA amounts between groups were held constant.

### Plasmids for transient transfections

The pRL Renilla Luciferase Control Reporter plasmid (Promega) and the PTHrP promoter plasmid were used as described [48]. The Gli2 promoter and protein reporter plasmids, as well the β-catenin plasmids were also a gift from Vladimir Spiegelman. The Smad2/3 protein plasmids were a gift from the laboratory of Dr. Harold Moses at Vanderbilt University.

### Quantitative real-time PCR

Cells were harvested by direct lysis and total RNA extracted using the RNeasy Mini Kit (Qiagen). The qScript cDNA synthesis kit (Quanta, VWR) was used to synthesize cDNA from 1ug RNA. Validated Taq-Man primers from (ThermoFischer Scientific) were used to measure gene expression in triplicate using the 7500 Real-Time PCR System from Applied BioSciences (ThermoFisher Scientific). Absolute gene expression was quantified using a standard curve and 18s was used as an internal control.

### Western blots

Briefly, cell lysate was harvested using RIPA Buffer (Sigma) supplemented with protease and phosphatase inhibitors (Roche). 20ug of protein were loaded per well and gels were run using Nu Page supplies (Novex by Life Technologies) before being transferred to PVDF membranes. Membranes were blocked in TBS with .1% Tween-20 and 5% BSA or 5% non-fat dry milk for 1 hour. Primary antibody incubations of GAPDH, Smad3 and phospo Smad3 (Cell Signaling) were done overnight at four degrees under gentle agitation, and the secondary antibody, anti-rabbit IgG (Cell Signaling) was incubated for one hour at room temperature under gentle agitation. Membranes were exposed on x-ray film using Western Lightening Plus-ECL (Perkin Elmer).

### Animal studies

All animal studies were carried out in compliance with the Vanderbilt University Institutional Animal Care and Use Committee and the National Institutes of Health guidelines.

#### Intra-tibial injections

5×10^5^ cells were injected into the left tibia of 4-6 week old athymic male mice from Harlan Laboratories. The right tibia was used as a PBS injection control. Weekly x-rays using the XR-60 digital radiography system from Faxitron were done to monitor tumor progression. At the end of the experiment, mice were sacrificed and hind limbs dissected for *ex vivo* analyses.

#### Masseter muscle injections

1×10^6^ cells were injected into the left masseter muscle (parallel to the mandible) of 4-6 week old athymic male mice from Harlan Laboratories. The right muscle was used as an injection control. Mice were weighed weekly to assess tumor burden. Drug treatments began once tumors were palpable (~10 days). At the end of the experiment, mice were sacrificed and mandibles were dissected for *ex vivo* analyses.

### Clinical OSCC samples

Dr. Kim Ely reviewed patient charts to identify OSCC patients that underwent a mandiblectomy as compared to those that underwent soft tissue removal, which was used to acquire 30 total OSCC samples on histological slides from the Translational Pathology Shared Resource Core at Vanderbilt. 16 available matching macrodissections were acquired from the Vanderbilt-Baker Head and Neck Bio-repository, and processed for qRT-PCR as described.

### Immunohistochemical staining

Mandible specimens were dissected and fixed in 10% neutral-buffered formalin (Fisher Scientific) for 48 hours at four degrees. Mandibles were then decalcified in 10% EDTA for 10 days at room temperature under agitation and embedded in paraffin. Mandible sections (5-7μm thickness) were stained with hematoxylin & eosin, orange G, and phloxine to measure tumor burden. Antibody staining against Gli2 (Novus Biologicals at 1:250) was used to measure Gli2 protein expression. Antibody staining against PTHrP (Jack Martin at 1:400) was used to measure PTHrP protein expression. Unlabeled goat IgG and rabbit IgG from Santa Cruz (1:400) were used as control primary antibodies. HRP linked rabbit anti-goat or goat anti-rabbit and ImmPACT NOVA RED from Vector Laboratories was used to detect staining. TRAP staining was used to measure osteoclast numbers. All slides were examined under an Olympus microscope at 20X and 40X and images (taken using Olympus DP71 camera and software) were quantified using Metamorph software (Molecular Devices, Inc.) for thresholding and region of interest (ROI) analysis.

### Immunohistochemical analyses

Histological images from HRP-labeled antibody staining were uploaded into Metamorph. For each image, the bottom incisor was used as a landmark. The area of tumor was traced using region of interest analyses, then, positive staining was quantified for each slide using a representative threshold (based on the positive control). For PTHrP, positive staining is marked by tumor specific brown staining. For Gli2, positive staining is marked by tumor specific nuclear brown staining. Non-specific staining from the IgG control was used measure background staining. This value was subtracted from each slide to normalize values. The resulting value represents positive staining, which is then divided by the total area of the image yielding percent positive staining. Similarly, tumor burden in tibiae were assessed using H&E staining, where tumor area is quantified and divided by the total area (total marrow space of tibia from the growth plate to near mid-shaft).

### Statistical analyses and replicates

All *in vitro* experiments were done in triplicate with a minimum n =3 samples. For intra-tibial injections, n=8 mice per group. For masseter muscle injections, n=10 mice per group. All statistical analyses were done using InStat v3.03 software from GraphPad Software. All values are presented as mean ± SEM where * denotes p<.05, ** denotes p<.01 and *** denotes p<.001.

## SUPPLEMENTARY FIGURES


