# Supplementary material for: Hedgehog and TGFβ signaling converge on Gli2 to control bony invasion and bone destruction in oral squamous cell carcinoma
Source: Oncotarget. 2016 Oct 12;7(46):76062–75. doi: 10.18632/oncotarget.12584 (PMC5340177; doi:10.18632/oncotarget.12584)
Supplement: Supplementary file 1 [file oncotarget-07-76062-s001.pdf]

Hedgehog and TGFβ signaling converge on Gli2 to control bony invasion and bone destruction in Oral squamous cell carcinoma

SUPPLEMENTARY FIGURES

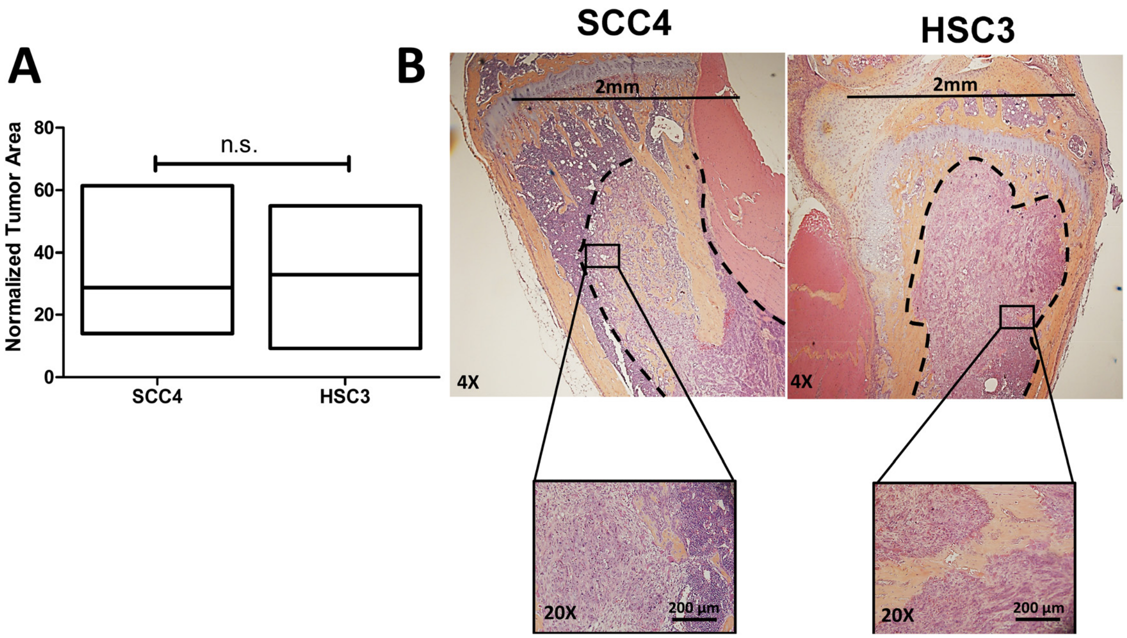

Supplementary Figure S1: SCC4 and HSC3 tumor burden is not significantly different in vivo. A. SCC4 and HSC3 tumors have comparable tumor area. SCC4 and HSC3 tumors cells injected into the tibia have tumor areas that are not statistically significant by histological analyses. **B.** Representative images of SCC4 and HSC3 tumor bearing tibias show similar tumor burden. 4X and 20X representative images of H&E stained tibia sections show similar tumor burden.

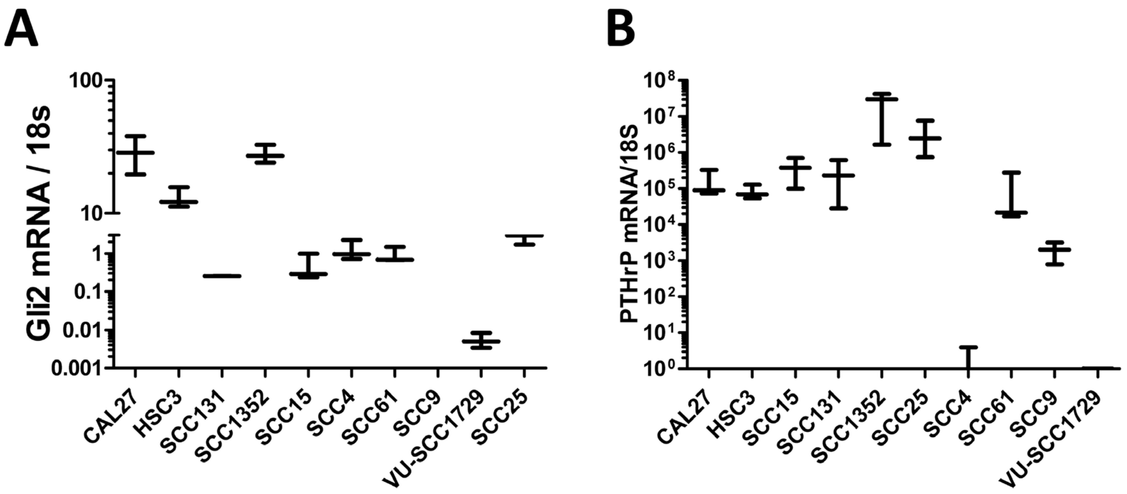

Supplementary Figure S2: Gli2 and PTHrP is expressed by nine of ten OSCC cell lines tested. A. OSCC express varying levels of Gli2. By qRT-PCR, all OSCC cell lines tested, except SCC9, expressed Gli2 mRNA at differing levels when kept in serum free conditions for 24 hours. **B.** OSCC express varying levels of PTHrP. By qRT-PCR, all OSCC cell lines tested, except VU-SCC1729, expressed PTHrP mRNA at differing levels when kept in serum free conditions for 24 hours.

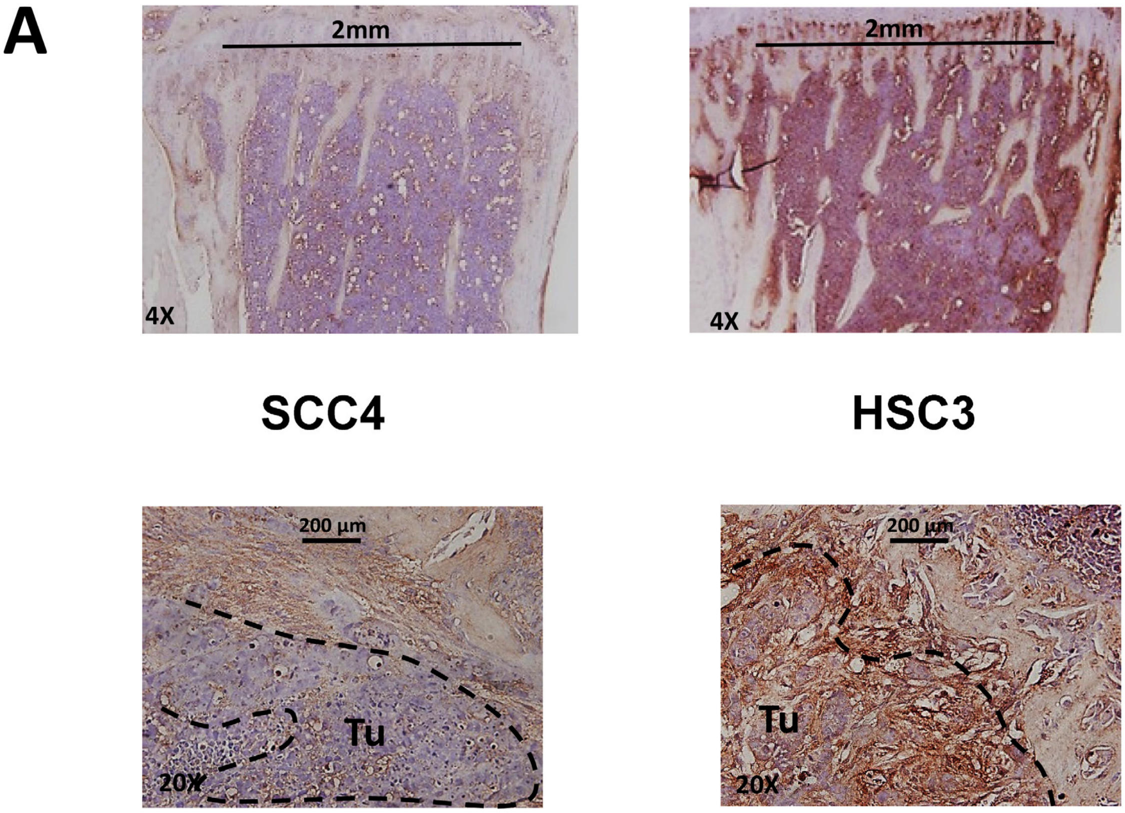

Supplementary Figure S3: Bony invasive status correlates with PTHrP levels *in vivo*. A. The bony invasive OSC cell line HSC3 express significantly more PTHrP protein than the non-bony invasive SCC4 OSCC cell line. Representative images (4X and 20X) of SCC4 or HSC3 cells injected into the tibia demonstrate different levels of PTHrP protein as shown by IHC staining. Black dotted lines indicate tumor front.

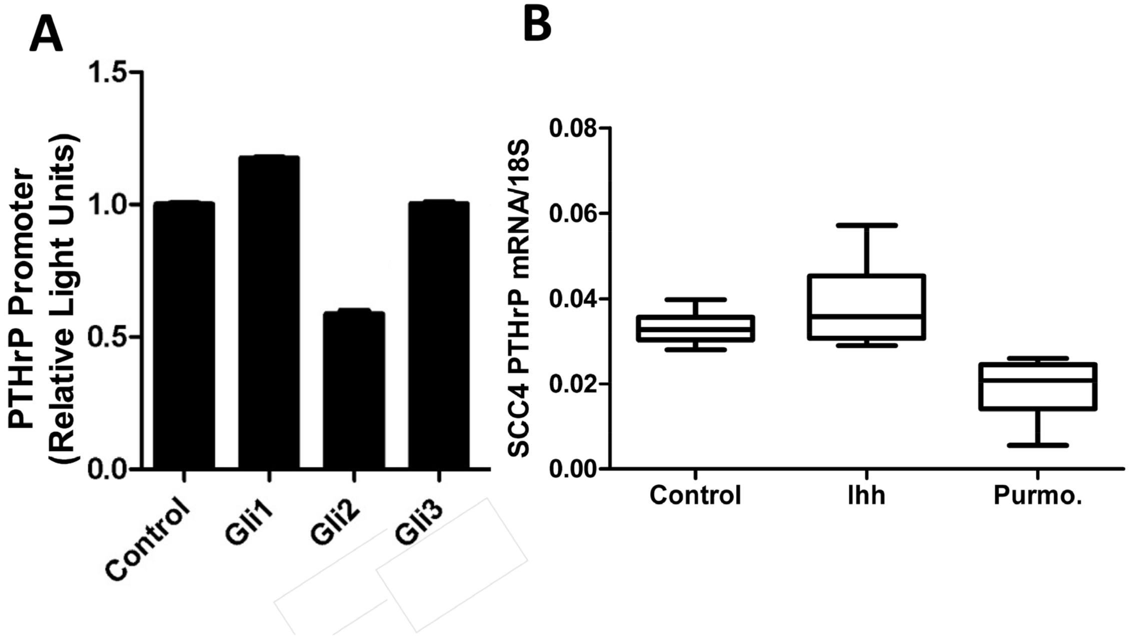

Supplementary Figure S4: Canonical Hh activation does not increase PTHrP expression levels. A. Gli protein overexpression does not increase PTHrP promoter activity. SCC4 cells transfected to overexpress Gli protein do not increase PTHrP promoter activity after 24 hours as shown by DLR assay. **B.** Exogenous Hh ligand does not increase PTHrP mRNA expression. SCC4 cells treated with ihh ligand or purmorphamine do not increase PTHrP mRNA expression after 24 hours of treatment.

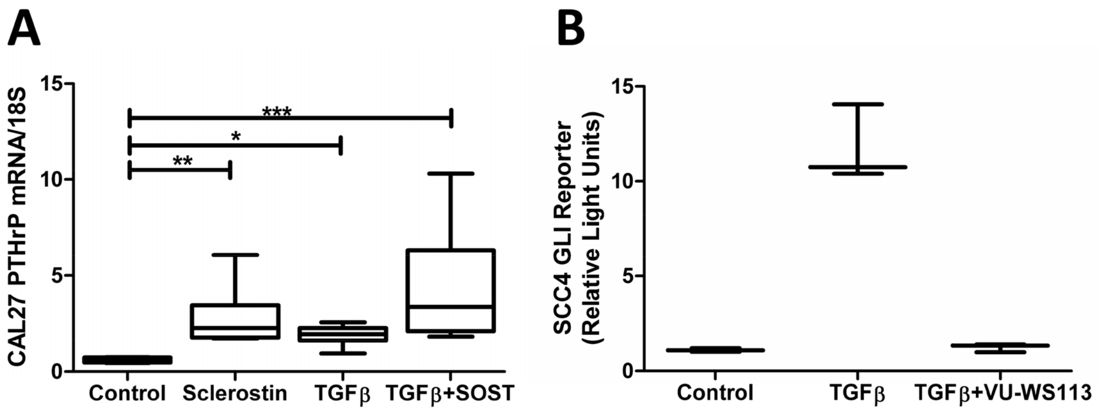

Supplementary Figure S5: Wnt signaling is dysregulated in OSCC and cross-talks with Hh/Gli signaling. A. Sclerostin treatment increase PTHrP expression. SCC4 cells treated with Sclerostin to inhibit Wnt signaling at the receptor level show increased levels of PTHrP mRNA after 24 hours of treatment, similar to TGFβ stimulation. **B.** CK1 inhibition decreases Gli activity. SCC4 cells treated with the small molecule Wnt inhibitor (VU-WS113) show decreased levels of PTHrP mRNA after 24 hours of treatment.

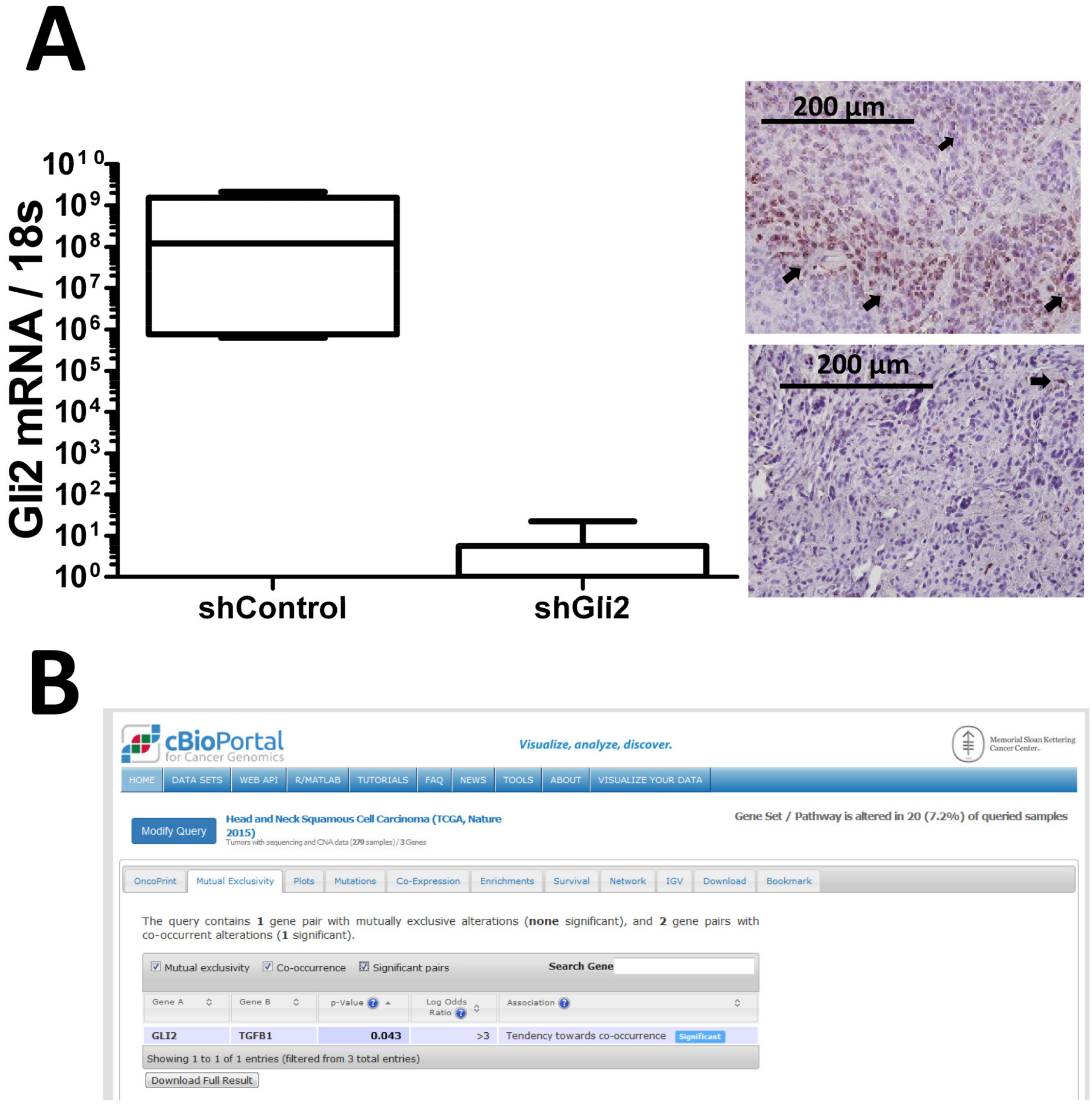

Supplementary Figure S6: Gli2 knock down tumors remain Gli2 negative in vivo and Gli2 and TGFβ significantly co-occur in HNSCC patients. A. shRNA against Gli2 significantly decrease Gli2 expression in OSCC. CAL27 cells transfected to stably express shRNA against Gli2 show significantly reduced levels of Gli2 mRNA by qRT-PCR. Representative images of CAL27 tumors show active Gli2 protein in control transfected cells and very little Gli2 protein in shGli2 transfected cells. (Positive staining is denoted by black arrows) **B.** Gli2 and TGFβ significantly co-occur in HNSCC patients Using the cBioPortal resource, a significant correlation was found between Gli2 and TGFβ1 expression in a cohort of 279 patients with head and neck cancer.
